# The role of short-chain dehydrogenase/oxidoreductase, induced by salt stress, on host interaction of *B. pseudomallei*

**DOI:** 10.1186/1471-2180-14-1

**Published:** 2014-01-02

**Authors:** Pornpan Pumirat, Usa Boonyuen, Muthita Vanaporn, Peechanika Pinweha, Sarunporn Tandhavanant, Sunee Korbsrisate, Narisara Chantratita

**Affiliations:** 1 Department of Microbiology and Immunology, Faculty of Tropical Medicine, Mahidol University, Bangkok 10400, Thailand; 2 Department of Molecular Tropical Medicine and Genetics, Faculty of Tropical Medicine, Mahidol University, Bangkok 10400, Thailand; 3 Department of Immunology, Faculty of Medicine, Siriraj Hospital, Mahidol University, Bangkok 10700, Thailand; 4 Mahidol-Oxford Tropical Medicine Research Unit, Faculty of Tropical Medicine, Mahidol University, Bangkok 10400, Thailand

## Abstract

**Background:**

*Burkholderia pseudomallei* is the causative agent of melioidosis, a frequently occurring disease in northeastern Thailand, where soil and water high in salt content are common. Using microarray analysis, we previously showed that *B. pseudomallei* up-regulated a short-chain dehydrogenase/oxidoreductase (SDO) under salt stress. However, the importance of SDO in *B. pseudomallei* infection is unknown. This study aimed to explore the function of *B. pseudomallei* SDO, and to investigate its role in interactions between *B. pseudomallei* and host cells.

**Results:**

Bioinformatics analysis of *B. pseudomallei* SDO structure, based on homology modeling, revealed a NAD^+^ cofactor domain and a catalytic triad containing Ser149, Tyr162, and Lys166. This is similar to *Bacillus megaterium* glucose 1-dehydrogenase. To investigate the role of this protein, we constructed a *B. pseudomallei* SDO defective mutant, measured glucose dehydrogenase (GDH) activity, and tested the interactions with host cells. The *B. pseudomallei* K96243 wild type exhibited potent GDH activity under condition containing 300 mM NaCl, while the mutant showed activity levels 15 times lower. Both invasion into the A549 cell line and early intracellular survival within the J774A.1 macrophage cell were impaired in the mutant. Complementation of SDO was able to restore the mutant ability to produce GDH activity, invade epithelial cells, and survive in macrophages.

**Conclusions:**

Our data suggest that induced SDO activity during salt stress may facilitate *B. pseudomallei* invasion and affect initiation of successful intracellular infection. Identifying the role of *B. pseudomallei* SDO provides a better understanding of the association between bacterial adaptation and pathogenesis in melioidosis.

## Background

*Burkholderia pseudomallei* is a Gram-negative bacillus and the causative agent of melioidosis, a severe disease endemic in Southeast Asia and northern Australia [1]. The organism is an environmental saprophyte found in soil and water. It infects humans and animals mostly by direct contact with wet soil [1,2]. The incidence of melioidosis is high in northeastern Thailand, where saline soil and water are abundant [3,4]. The salt concentration in soil in this region ranges from 40 to 1,000 mM NaCl – significantly higher than the 20 mM NaCl average in other parts of the country (Development Department, Ministry of Interior, Thailand). It has been suggested that high salt or osmotic stress in northeast Thailand may be a key factor for *B. pseudomallei* alteration for survival in the natural environment, and it may enable the bacteria to establish the infection in respective hosts. The relationship between high salt concentration and susceptibility to bacterial infection is described in cystic fibrosis (CF) patients [5]. The lung airway surface liquid of CF sufferers has twice the NaCl concentration of healthy lungs [6]. Opportunistic infections of CF lungs have been linked with a variety of pathogens, including *B. cepacia* complex [7,8] and *B. pseudomallei*[9]. However, the impact of salt and osmotic stress on *B. pseudomallei* and the related mechanisms underlying *B. pseudomallei* pathogenesis in CF patients are unknown.

An earlier study demonstrated that the killing efficiency of *Burkholderia* species, including *B. pseudomallei*, against the nematode *Caenorhabditis elegans* is enhanced in condition containing 300 mM NaCl [10]. We also showed that *B. pseudomallei* grown under salt stress invades a lung epithelial cell line A549 [11] more efficiently, and exhibits significantly greater resistance to ceftazidime, an antibiotic used to treat melioidosis [12]. Our transcriptional analysis revealed *B. pseudomallei* pre-exposed to salt stress up-regulates a 10-fold increase of a gene associated with short-chain dehydrogenase/oxidoreductase (SDO) [11]. A different study by Bhatt & Weingart [13] also showed that an oxidoreductase encoding gene (*bsrA*) was up-regulated in *B. cenocepacia* in response to increased NaCl concentrations. However, the role of SDO for *B. pseudomallei* adaptation to osmotic or salt stress remains unknown.

In the present study, we analyzed the protein sequence and predicted structure of *B. pseudomallei* SDO using bioinformatics analysis, to provide information about the possible functions of SDO. We further investigated its functional roles by constructing a SDO deletion mutant strain, and examined the interaction between mutant and host cells. The results suggest that SDO is an adaptive determinant of *B. pseudomallei* virulence, which is up-regulated under salt stress, and has a significant role in the interaction with host cells.

## Results

### Bioinformatics analysis of *B. pseudomallei* SDO

**Figure 1:**
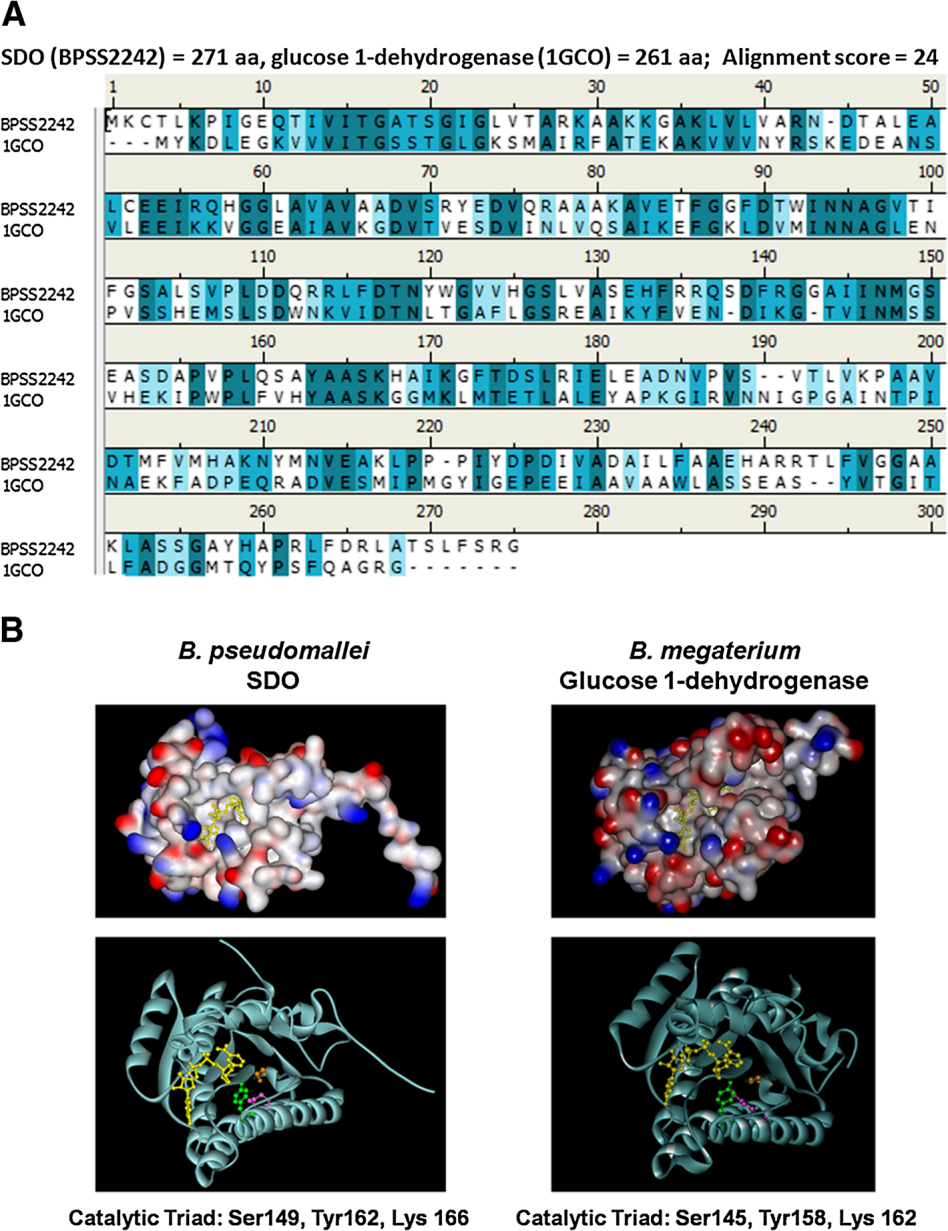
**Protein sequence and structural comparison between *B. pseudomallei *SDO and *B. megaterium *glucose 1-dehydrogenase. A)** Sequence alignment between *B. pseudomallei* SDO and *B. megaterium* glucose 1-dehydrogenase. **B)** Structural model of *B. pseudomallei* SDO (left) and structure of *B. megaterium* glucose 1-dehydrogenase (right), with bound NAD (yellow) shown in both surface (top) and cartoon representations (bottom). *B. pseudomallei* SDO and *B. megaterium* glucose 1-dehydrogenase shared structural similarities with conserved catalytic triad, consisting of Tyr (green), Thr (pink) and Lys (orange). Figures were generated by Discovery Studio Visualizer – Accelrys.

A SDO amino-acid (aa) sequence of *B. pseudomallei* strain K96243 was retrieved from GenBank (NCBI Reference Sequence: YP_112245.1; locus_tag = “BPSS2242” [14]). It was composed of 271 aa with a calculated molecular weight of 28,766 Dalton. BLAST [15] sequence analysis [16] revealed that *B. pseudomallei* SDO was categorized into short-chain dehydrogenases/reductases (SDRs), which shared a 24% amino-acid sequence identity with *Bacillus megaterium* glucose 1-dehydrogenase (PDB ID: 1GCO) (Figure 1A). Therefore, the SWISS-MODEL [17] was used to construct a structural model of *B. pseudomallei* SDO, using *B. megaterium* glucose 1-dehydrogenase as a template for homology modeling. The resulting model was validated by PROCHECK [18]. The structural model of *B. pseudomallei* SDO revealed a catalytic triad active site, consisting of Ser149, Tyr162, and Lys166, together with a NAD^+^ cofactor domain (Figure 1B). This suggests that the SDO of *B. pseudomallei* may have an enzymatic function similar to *B. megaterium* glucose 1-dehydrogenase.

Among available genomes of *Burkholderia* spp., BLAST analysis demonstrated that all species harbor the SDO protein. The amino-acid identities of pathogenic *B. pseudomallei*, *B. mallei*, *B. oklahomensis*, *B. multivorans*, *B. vietnamiensis*, and *B. cenocepacia* range from 83% to 100%, whereas those of non-pathogenic *B. thailandensis* are less than 36%. The high identity among pathogenic strains might indicate a common pathogenesis that is mediated by *Burkholderia* SDO.

### Mutagenesis of *B. pseudomallei* SDO mutant

To identify the function of SDO in *B. pseudomallei*, we constructed a mutant defective in SDO production using a pEXKm5-based allele replacement system [19]. PCR analysis using primers flanking deleted alleles confirmed the deletion of the SDO gene on the *B. pseudomallei* chromosome (Additional file 1). As expected, a 566 bp DNA fragment was detected in the SDO mutant, whereas a 1,197 bp DNA fragment was detected in the wild type K96243, indicating a homologous recombination by deletion of 631 bp of the SDO gene on the chromosome of the *B. pseudomallei* mutant. *B. pseudomallei* SDO complement strain was constructed using the same strategy. The amplified product of the complemented strain showed the presence of a 1,197 bp DNA fragment, as observed in the parental strain. PCR analysis of a 236 bp oriT fragment demonstrated an extinction of pEXKm5 plasmid backbone in both the mutant and complement strains.

The pEXKm5 plasmid was removed from the SDO mutant and the complement strains by sucrose selection. Absence of a 236 bp oriT amplicon indicated the removal of pEXKm5 plasmid from the chromosome of the *B. pseudomallei* SDO mutant and the complement strains.

### *B. pseudomallei* SDO exhibits GDH activity under salt stress

*B. pseudomallei* is known to up-regulate SDO in high salt condition [11]. The structural model of *B. pseudomallei* SDO indicates a catalytic triad and cofactor binding domain, similar to the structure of *B. megaterium* glucose 1-dehydrogenase. This is highly specific to beta-D-glucose and is capable of using either NAD^+^ or NADP^+^ as a cofactor [20]. We hypothesized that the glucose dehydrogenase activity of *B. pseudomallei* SDO might be similar to *B. megaterium.* We determined the GDH activity of *B. pseudomallei* SDO in wild type and SDO mutant strains grown in LB broth containing 0–300 mM NaCl. The results showed that *B. pseudomallei* wild type exhibited strong GDH activity under high salinity at 300 mM NaCl, whereas the activity of *B. pseudomallei* was comparable in salt-free and 150 mM NaCl (Table 1). This correlated with previous finding that suggested *B. pseudomallei* SDO transcription was enhanced by salt stress [11].

**Table 1:** **Effect of NaCl treatment on GDH activity by *B. pseudomallei *** **K96243, SDO mutant, and complement strains**

**NaCl**	**GDH activity mU/mg**		
**(mM)**	**K96243**	**SDO mutant**	**SDO complement**
0	0.049 ± 0.006	0.045 ± 0.003	0.042 ± 0.005
150	0.066 ± 0.012	0.050 ± 0.027	0.056 ± 0.017
300	0.996 ± 0.109	0.067 ± 0.026	0.952 ± 0.060

Data represent mean ± standard error (SE) of three experiments made in triplicate.

It was also evident that the GDH activity of SDO mutant was impaired under high salt concentration condition containing 300 mM NaCl (Table 1), which was 15-fold lower than the wild type (*p*-value ≤ 0.0001). The SDO complement strain was able to recover SDO mutant GDH activity (Table 1). The data suggested that high salt concentration is associated with induction of SDO-dependent GDH activity in *B. pseudomallei*.

### SDO plays a role in host interaction of *B. pseudomallei*

**Figure 2:**
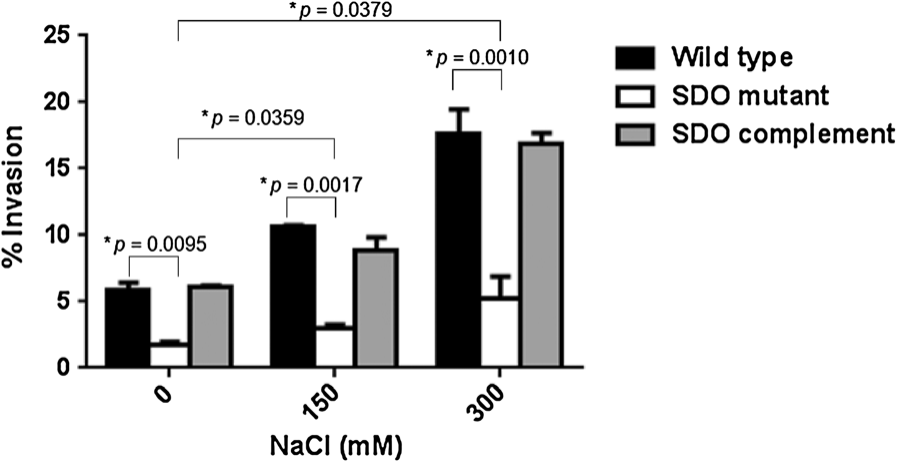
**Invasion of A549 epithelial cells by *B. pseudomallei.*** A549 cells were infected with overnight cultures of *B. pseudomallei* K96243 at MOI of 100, SDO mutant, and complement strains grown in NaCl-free LB broth, LB broth with 150 mM NaCl, or LB broth with 300 mM. Intracellular bacteria were counted after lysing infected cells at 4 hrs post-infection. Asterisks indicate significant differences (*p*-value ≤ 0.05, *t*-test) between groups. Error bars represent standard errors of mean for experiments performed in triplicate.

The ability of *B. pseudomallei* to invade and survive in host cells is an important process that contributes to the pathogenesis of melioidosis. Invasion of *B. pseudomallei* has been reported as being induced by exogenous salt [11], and previous study indicated that high salt concentration increases the expression of SDO [11]. We thus investigated whether SDO affects the invasion of *B. pseudomallei* into A549 human lung respiratory epithelial cells. We found that invasion efficiency into A549 cells was significantly reduced in the *B. pseudomallei* SDO mutant, compared to the wild type (*p*-value ≤ 0.05) (Figure 2). The invasion efficiency of the *B. pseudomallei* mutant grown in LB broth containing 0, 150, and 300 mM NaCl was less than 5%, in contrast to that of wild type bacteria (5.90%, 10.57%, and 17.68%, in LB with 0, 150, and 300 mM NaCl, respectively). While the mutant had less invasion efficiency, the result clearly demonstrated that increasing salt concentration from 0 to 150 or 300 mM NaCl led to significantly improved invasion of *B. pseudomallei* mutant into A549 cells as it is observed for the wild type strain (Figure 2).

**Figure 3:**
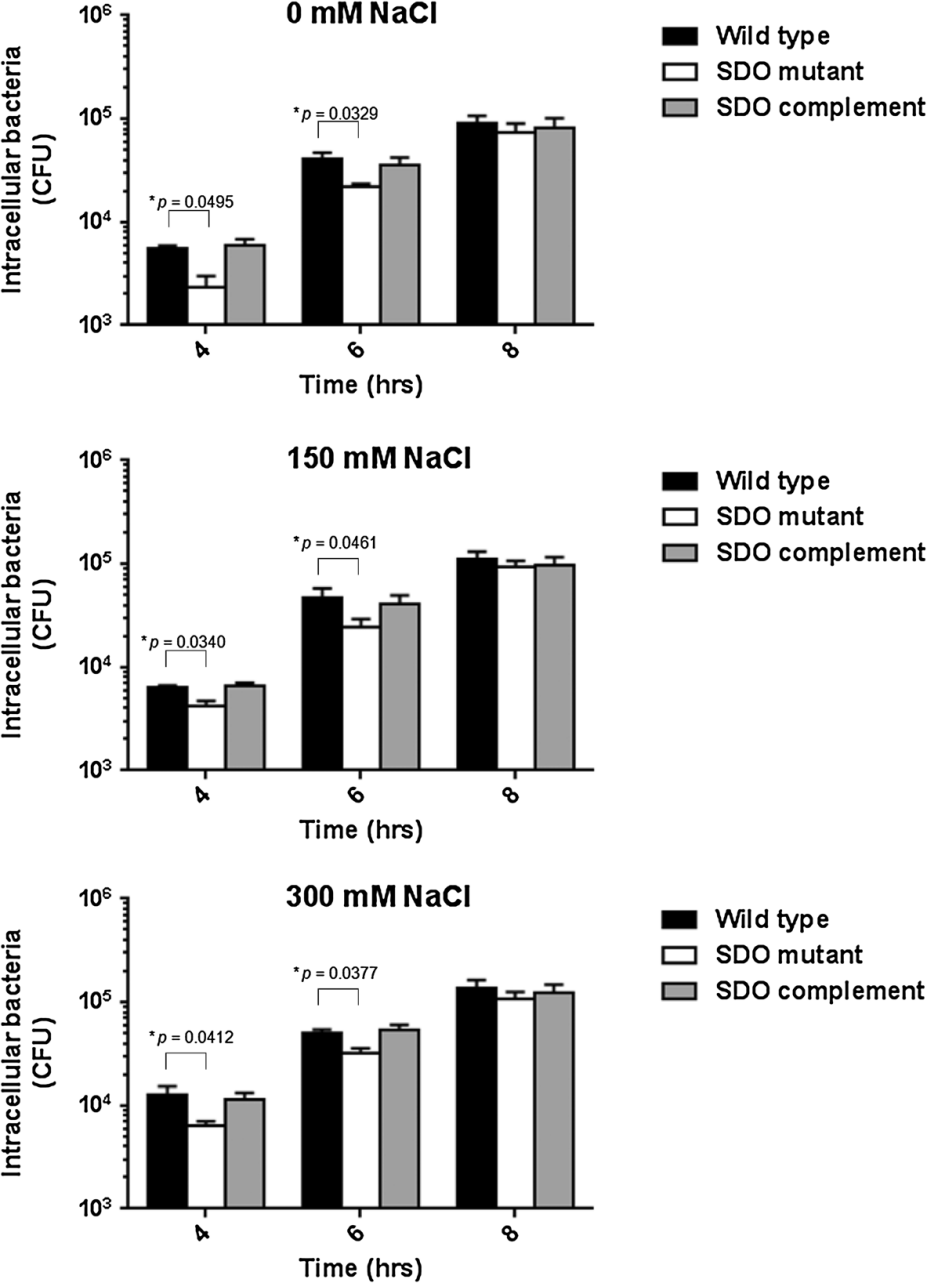
**Intracellular survival of *B. pseudomallei *in J774A.1 macrophages.** J774A.1 cells were infected with overnight cultures of *B. pseudomallei* K96243 at MOI of 2, SDO mutant and complement strain grown in NaCl-free LB broth, LB broth with 150 mM NaCl, or LB broth with 300 mM. Intracellular bacteria were counted after lysing infected cells at 3, 6 and 9 hrs post-infection. Asterisks indicate significant differences (*p*-value ≤ 0.05, *t*-test) between groups. Error bars represent standard errors of mean for experiments performed in triplicate.

The ability of *B. pseudomallei* to survive and replicate intracellularly may be attributable to the successful evasion of cellular killing strategies. We next examined the intracellular survival of the *B. pseudomallei* wild type and the SDO mutant within macrophages. The macrophage cells were chosen for this experiment because *B. pseudomallei* can be uptaken and multiply within these cells, and resist their bactericidal response [21,22]. The mutant showed fewer intracellular bacteria within the J774A.1 macrophage cell line during the initial stages of infection – up to 6 hrs (*p***-**value ≤ 0.05) (Figure 3). The intracellular doubling time of the *B. pseudomallei* SDO mutant pre-exposure to 0, 150, and 300 mM NaCl was 41.83 ± 1.71, 45.41 ± 2.66, and 50.41 ± 1.33%. In contrast, the doubling time of the wild type bacteria was 32.50 ± 4.29, 36.39 ± 1.44, and 47.23 ± 2.31% in LB with 0, 150, and 300 mM NaCl. The SDO complement strain recovered the growth of the SDO mutant with a rate similar to the wild type at an early time. Our data suggests that SDO plays an important role during the early phase of *B. pseudomallei* infection. It is possible the mutagenesis of SDO impaired the invasion of *B. pseudomallei* into A549 epithelial cells, and delayed initial multiplication within J774A.1 macrophage cells.

### SDO is not essential for *B. pseudomallei* growth and colony morphotype

**Figure 4:**
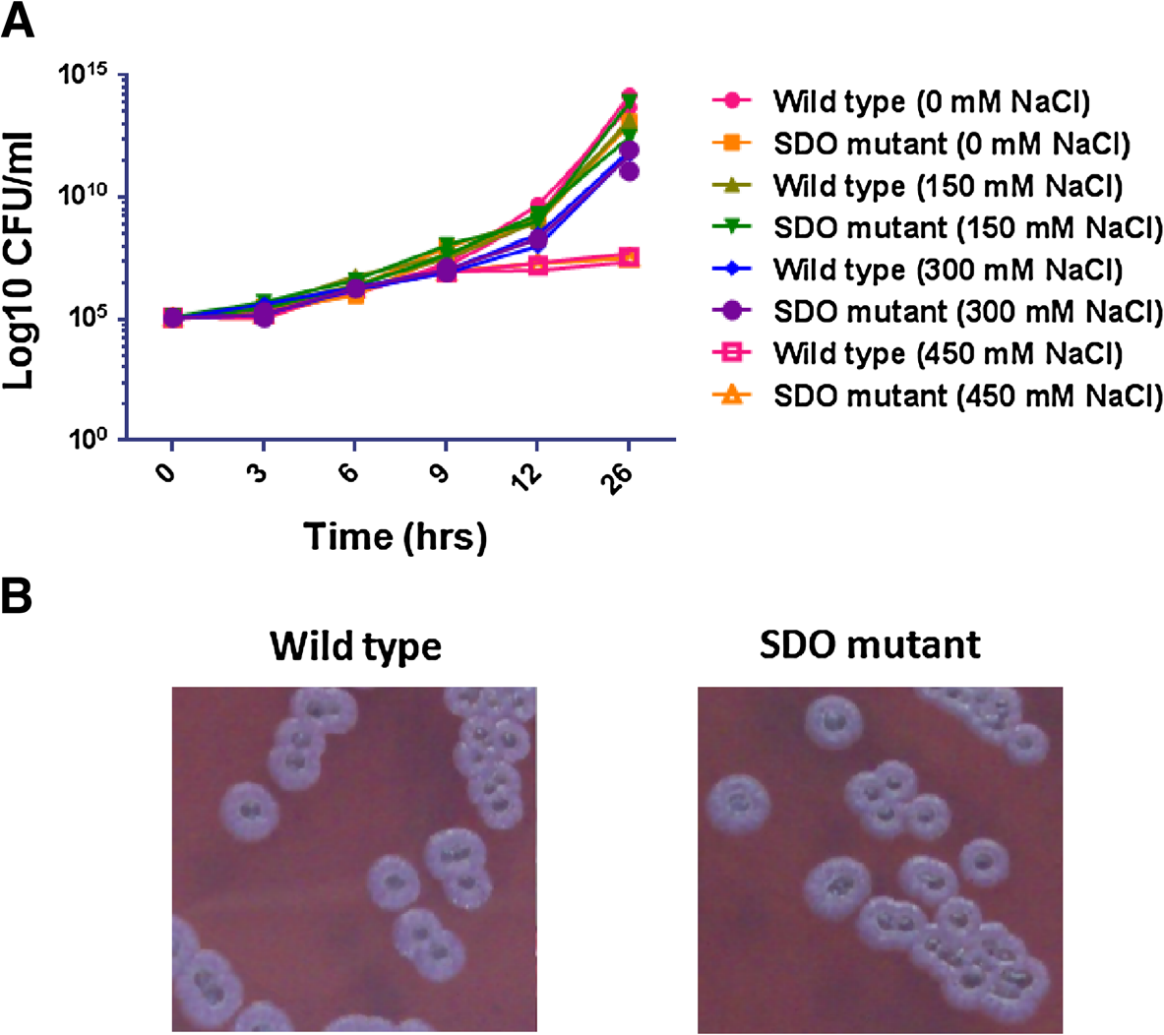
**Growth kinetics of *B. pseudomallei. *A)***B. pseudomallei* K96243 and SDO mutant growth in LB broth containing 0, 150, 300 or 450 mM NaCl was determined by colony plate counting. The data points and error bars represent mean and standard deviation from triplicate experiments. **B)***B. pseudomallei* K96243 and SDO mutant growth on Ashdown agar for 4 days. The colony morphology was examined using a morphotyping algorithm [26].

Some dehydrogenases are critical for the growth characteristic of bacteria [23-25]. We investigated the effect of SDO deletion on the growth of *B. pseudomallei*. Growth of the wild type K96243 and the SDO mutant was compared in Luria-Bertani (LB) medium, containing various concentrations of NaCl (0, 150, 300, and 450 mM). We observed that the growth kinetics of the *B. pseudomallei* K96243 and the SDO mutant were comparable (Figure 4A). The culture condition containing 450 mM NaCl impaired the growth of both strains. Variations in colony morphology are a notable feature of *B. pseudomallei* growth, where certain types are associated with enhanced bacterial survival under adverse conditions [26]. We also examined the effect of SDO on colony morphotype switching in the *B. pseudomallei* K96243 and the SDO mutant on Ashdown agar. The results indicated no phenotypic change of colony morphology between the wild type K96243 and the mutant. Both were categorized as colony morphotype I [26] (Figure 4B). These results indicated that SDO deletion does not affect *B. pseudomallei* colony morphology and bacterial growth.

### SDO is not required for *B. pseudomallei* survival under oxidative stress

Many reports suggested that dehydrogenases are associated with the bacterial protection against toxic oxidants [27-33]. We examined the role of SDO for survival of *B. pseudomallei* under different oxidative stress conditions. Salt-treated and untreated *B. pseudomallei* wild type and SDO mutant strains were cultured on LB agar plates containing 250 μM H_2_O_2_, 400 μM menadione, or 200 μM tert-butyl hydroperoxide (tBOOH), and their survival were determined (Table 2). The result showed that there are no significant differences in survival between the *B. pseudomallei* wild type and the SDO mutant strains, neither in salt-treated or untreated conditions. This indicates that SDO might not be essential for adaptation and growth of *B. pseudomallei* in these oxidative stress environments.

**Table 2:** **Effect of NaCl treatment on *B. pseudomallei *** **survival under oxidative stress conditions**

***B. pseudomallei***	**NaCl (mM)**	**% Bacterial survival**			
		**Control**	**250 μM H**_**2**_**O**_**2**_	**400 μM menadione**	**200 μM tBOOH**
K96243	0	100	58.6 ± 4.3	17.2 ± 3.7	62.6 ± 2.4
	150	100	75.8 ± 2.6	31.0 ± 3.4	65.4 ± 3.3
	300	100	82.8 ± 3.9	72.4 ± 4.7	68.9 ± 5.5
SDO mutant	0	100	60.9 ± 3.4	17.8 ± 2.9	58.5 ± 2.4
	150	100	72.7 ± 4.0	32.7 ± 5.8	64.0 ± 3.9
	300	100	86.2 ± 5.1	75.8 ± 6.2	67.6 ± 5.5

Data represent mean ± SE of three experiments made in triplicate.

## Discussion and conclusions

*B. pseudomallei* is a saprophyte bacterium habitats in environments in endemic area, including Northeast Thailand [1] where saline salt and water are abundant. In normal physiological conditions, the NaCl concentration in the human lung is between 50 to 100 mM, and in the blood it can be as high as 150 mM [34,35]. In CF patients, the defective lung airway surface liquid has twice the NaCl concentration compared to healthy lungs [6,34]. It has been reported that elevated salt levels causes failure of bacterial killing in CF patients [5,6,34]. The opportunistic infection of CF lungs is linked to a variety of pathogens, including *B. pseudomallei*[7-9]. There is increasing evidence suggesting that salt concentration or osmolarity in a habitat influences the survival and pathogenicity of *B. pseudomallei*[10-12,36,37]*.* Thus, understanding the effect of salt stress is beneficial not only for environmental adaptation but also pathogenesis of the disease.

To survive in a high salt environment, the bacteria can undergo adaptation by altering the regulation of gene expression. Using transcriptomic analysis, we recently discovered that *B. pseudomallei* responds to salt stress by modulating the transcription of specific genes [11]. Among these are several loci associated with unknown functions, which need to be identified. Changes of *B. pseudomallei* transcriptome under salt stress include increasing expression of SDO [11]. The SDO is an enzyme in the short-chain dehydrogenases/reductases/oxidoreductase family that catalyzes the following chemical reaction: D-glucose + NAD^+^ = D-glucono-1,5-lactone + NADH + H^+^. Both NADP^+^ and NAD^+^ are usually utilized as cofactors [38]. This study revealed the importance of SDO expression during salt-stress adaptation. Based on the structural model of *B. pseudomallei* SDO, which consists of a NAD^+^ cofactor domain and catalytic triad containing Ser149, Tyr162, and Lys166 similar to *Bacillus megaterium* glucose 1-dehydrogenase, we hypothesized that *B. pseudomallei* SDO has GDH activity. To examine the function of *B. pseudomallei* SDO, a mutant strain lacking SDO was constructed using a gene replacement strategy, a method that rarely has a polar effect on downstream genes [19]. In contrast to the wild type, it is clear that the *B. pseudomallei* SDO mutant was unable to produce GDH activity under high salt concentration. This finding is consistent with our previous observation of transcriptome profiling that *B. pseudomallei* grown in LB broth with 320 mM NaCl induced a 10-fold up-regulation of the SDO gene [11]. Since the mutant lost the gene encoding for functional SDO enzyme, it was thus unable to catalyze the reaction.

Several studies indicate that dehydrogenase enzymes are critical for bacterial growth. For instance, Brown & Whiteley [23] have shown that the gene AA02749 (*lctD*), encoded for an NAD^+^-independent L-lactate dehydrogenase, is necessary for the growth of *Aggregatibacter actinomycetemcomitans*. Inactivation of the AA02769 gene affects the growth of the bacteria in the presence of L-lactate. Another study showed that mutations of the *nuo* gene encoding for NADH dehydrogenase I affected *E. coli* growth during the stationary phase culture in tryptone broth [24]. In our current study, we found that the *B. pseudomallei* mutant lacking SDO had growth kinetics and colony phenotypes similar to the *B. pseudomallei* wild type. At various salt concentrations, there was no significant difference in growth between both *B. pseudomallei* strains. It indicated that deletion of the SDO gene has no effect on *B. pseudomallei* growth. This result is in agreement with previous observations identified by microarray analysis – the SDO gene is not in a group of growth-phase regulated genes [39].

The association between dehydrogenase enzymes and bacterial pathogenesis has been reported in several studies [40,41]. The alcohol acetaldehyde dehydrogenase (lmo1634), also known as *Listeria* adhesion protein, which is present in pathogenic *Listeria* species, mediates pathogenicity by promoting bacterial adhesion to enterocyte-like Caco-2 cells [42]. It was shown that both lipoamide dehydrogenase “Lpd”, a member of three multienzyme complexes in pyruvate dehydrogenase complex, and 3-ketosteroid 1(2)-dehydrogenase are important for virulence of *Mycobacterium tuberculosis*[43,44]. In *Pseudomonas aeruginosa*, the SDO attenuated mutant had significantly reduced pyocyanin production, motility, and biofilm formation, as well as absent paralysis of *C. elegans*[45]. Consistent with these reports, our study shows that defective SDO is associated with a reduced efficiency of the mutant to invade into A549 lung epithelial cells. Furthermore, we observed that the invasion of the *B. pseudomallei* SDO mutant was enhanced by increasing concentration of NaCl to 150 or 300 mM. Compared to the wild type, the SDO mutant exhibited fewer invasions and subsequently revealed less replication at early infection time point, but at 8 hrs after infection the mutant was able to multiply in J774A.1 macrophage cells. The results suggest that the SDO gene might be induced only upon bacterial invasion of macrophage. It should be noted that *B. pseudomallei* grown under high salt conditions *in vitro* can up-regulate other virulence genes such as *bsa* T3SS. It is possible that this increased invasion was partly controlled by other salinity associated invasion- and virulence mechanisms, at least by coordinating regulation of the *bsa* T3SS [11]. Previous studies have demonstrated that the mutant defect in *bsa* T3SS genes such as *bsaZ* and *bipD* remained trapped in vesicles at earlier infection time points, but at 8 and 12 hrs after infection, the *bsaQ* and *bsaZ* mutants are able to escape into the cytosol and multiply effectively [46,47]. However, our finding in this study indicates that the SDO is involved in the pathogenesis of *B. pseudomallei* by facilitating the invasion and initial intracellular survival within host cells. It is feasible that SDO modulates the NAD^+^- or NADP^+^-dependent reaction associated with virulence expression when the *B. pseudomallei* encounters high salt stress.

The central role of bacterial defense against oxidative stress has been reported in many pathogenic bacteria [30,48,49], especially during aerobic respiration and interactions with phagocytic cells. Several reports have indicated that bacterial dehydrogenases are important enzymes in oxidative stress response, such as NADH dehydrogenase, lactate dehydrogenase, formate dehydrogenase, succinate dehydrogenase, fumarate reductase, and glutathione-dependent formaldehyde dehydrogenase [27-32]. In *Bacillus subtilis*, two glucose dehydrogenases (YxnA and YcdF) assigned to a family of short-chain dehydrogenases are required for severe ethanol stress [33]. In our present study, we found no difference in bacterial counts between the SDO mutant compared to the wild type *B. pseudomallei* on LB agar plates containing various oxidative agents for both NaCl-treated and untreated conditions. This indicates that SDO might not be crucial for *B. pseudomallei* to survive in oxidative stress environments. However, the survival under oxidative stresses increased in NaCl-treated *B. pseudomallei* with higher concentrations, from 0 mM to 150 mM, and up to 300 mM NaCl (Table 2). This finding suggests that NaCl may contribute to increase the oxidative stress tolerance of *B. pseudomallei.* Understanding the mechanism linking *B. pseudomallei* adaptation in saline environments to oxidative resistance requires further investigation.

In conclusion, our study revealed that *B. pseudomallei* SDO is involved in enhanced GDH activity in salt stress environments. The *B. pseudomallei* mutant lacking SDO had reduced abilities in invasion and initial intracellular survival. This indicates that this enzyme is associated with the pathogenesis of *B. pseudomallei*, especially when *B. pseudomallei* encounter salt stress. Due to the important role of SDO in pathogenesis, microbial SDOs might be a new target for the development of novel antibiotics. Thus, an understanding of the salt stress response of *B. pseudomallei* by the induction of SDO may provide important information in developing a new strategy for treatment of melioidosis.

## Methods

### Bacterial strains, growth conditions, and cell lines

*B. pseudomallei* wild type (K96243), the SDO mutant, and the complement strains were cultured in Luria-Bertani (LB) medium and grown at 37°C.

*B. pseudomallei* growth kinetics under stress conditions were performed as previously described [11]. The overnight culture of *B. pseudomallei* adjusted to OD_600_ 0.5 was inoculated 1:500 into 10 ml of LB broth, with or without NaCl (Merck). Every 2 hrs after inoculation, the optical density of cultures at various time points was recorded, and serial dilution of these cultures was performed for colony-forming unit counts (CFU).

The cell lines A549 (human respiratory epithelial cell) and J774A.1 (mouse macrophage cell) were maintained in Dulbecco’s Modified Eagle Medium (DMEM), which was supplemented with 10% heat-inactivated (30 min, 56°C) fetal bovine serum (FBS), and incubated at 37°C in a humidified incubator in the presence of 5% CO_2_.

### Bioinformatics analysis of *B. pseudomallei* SDO

The *B. pseudomallei* SDO amino-acid sequence was subjected to basic local alignment search (BLAST) [15]; further alignment was then performed using ClustalW [16]. The sequence with maximum identity, *Bacillus megaterium* glucose 1-dehydrogenase, was used as a template for homology modeling using SWISS-MODEL [17]. The constructed model was validated by PROCHECK [18].

### Construction of *B. pseudomallei* SDO deletion mutant and complemented strain

Deletion mutagenesis of the SDO gene was performed by homologous recombination (Additional file 1), as previously described by Lopez et al. [19]. The *B. pseudomallei* K96243 SDO gene sequence was obtained from GenBank (accession number NC_ 006351 and locus_tag = “BPSS2242” [14]). Primers used in this study were designed using Primer-BLAST (http://www.ncbi.nlm.nih.gov/tools/primer-blast). The primer sequences are shown in Table 3. Molecular cloning was carried out on 5′ 298 bp upstream and 3′ 288 bp downstream fragments of the *B. pseudomallei* SDO gene. The 5′ upstream and 3′ downstream fragments of the SDO gene were ligated by PCR using BPSS2242-F1 and BPSS2242-R2; this was facilitated by a tail on the 3′ forward primer to give a new PCR product with a deletion in the region (631 bp) between BPSS2242-R1 and BPSS2242-F2.

**Table 3:** Oligonucleotide primers used for PCR

**Primer names**	**Oligo sequences (from 5′–3′)**	**Purpose**	**Reference**
BPSS2242-F1	ACCGCGCGACCGATATGAACG	Forward primer for upstream fragment of SDO gene	This study
BPSS2242-F2	GGACTCCTTGCCGAACGGGC	Reverse primer for upstream fragment of SDO gene	This study
BPSS2242-R1	GCCCGTTCGGCAAGGAGTCC AACGTCGAGGCGAAGCTGCC	Forward primer for downstream fragment of SDO gene	This study
BPSS2242-R2	TCCCTTCGCGCTCGTGCAAC	Reverse primer for downstream fragment of SDO gene	This study
OriT-F	CAGCCTCGCAGAGCAGGATTC	Forward primer for *oriT*	[50]
OriT-R	TCCGCTGCATAACCCTGCTTC	Reverse primer for *oriT*	[50]

This constructed fragment was cloned into pGEM®-T Easy Vector and transformed into *Escherichia coli* strain DH5α. White colonies were selected using β-galactosidase indicator medium, using 50 μg/ml 5-bromo-4-chloro-3-indolyl-β-D-galactopyranoside (X-Gal) (Promega) plates containing 100 μg/ml ampicillin. Colonies harboring the desired plasmid were analyzed by PCR using primers flanking the mutant allele (BPSS2242-F1 and BPSS2242-R2). Products were checked for correct size by agarose gel electrophoresis and verified by DNA sequencing.

The unmarked knockout cassette assembled by PCR containing the deletion of the SDO gene was cloned into the non-replicative plasmid, pEXKm5 [19]. The pEXKm5-mutant allele construct was then transformed into *E. coli* strain DH5α. Plasmids were extracted and checked by PCR, with primers BPSS2242-F1 and BPSS2242-R2, for correct product sizes of the target gene. The pEXKm5-mutant plasmid was transformed into *E. coli* strain RHO3 and delivered to the host *B. pseudomallei* strain K96243 by conjugation. This resulted in integration of the allelic replacement construct into the *B. pseudomallei* chromosome by homologous recombination between cloned and chromosomal sequences.

Conjugant clones grown on LB agar containing 1000 μg/ml kanamycin and 50 μg/ml 5-bromo-4-chloro-3-indolyl-β-D-glucuronide (X-Gluc) (Promega) were selected for PCR, with primers flanking the mutant allele (BPSS2242-F1 and BPSS2242-R2).

The conjugant clones were then streaked onto yeast extract tryptone (YT) agar (Yeast Extract & Tryptone, BD; Agar, Oxoid) containing 15% sucrose and 50 μg/ml X-Gluc, and incubated at 25°C for 72 hrs. The colonies growing on X-Gluc-containing medium (YT-sucrose-X-Gluc plate) were selected and purified by streaking on the same medium, and incubated as described above. Confirmation of deletion mutant was performed by PCR using primer sets flanking the mutant deletion allele primers (BPSS2242-F1 and BPSS2242-R2) and the oriT pEXKm5 plasmid backbone sequences.

Complement strains were constructed using the same pEXKm5-based allele replacement approach. Forward and reverse primers corresponding to the relevant regions of the genome sequences were amplified by BPSS2242-F1 and BPSS2242-R2 primers. The PCR amplicon (1,197 bp) contained the wild type *B. pseudomallei* SDO sequence. The construct was cloned into pEXKm5, transformed into *E. coli* RHO3, and delivered to the *B. pseudomallei* mutant by conjugation, resulting in merodiploid formation. Sucrose selection was employed for merodiploid resolution, resulting in the generation of wild type sequences, as well as strains that maintained the deletion alleles. PCR was performed with primers flanking deleted alleles to screen for strains that had the mutant allele replaced with the wild type sequence. PCR with oriT-specific primers [50] was used to demonstrate the absence of pEXKm5 plasmid backbone.

### GDH activity assay

An overnight culture of *B. pseudomallei* wild type K96243, SDO mutant, and complement strains grown in salt-free LB broth, was subcultured 1:10 into LB broth containing 0, 150, or 300 mM NaCl and incubated at 37°C for 6 hrs. The bacteria cells were then examined by OD600 measurement and CFU plate counting, to confirm that they derived from cultures containing the same numbers of viable bacteria. *B. pseudomallei* wild type K96243, SDO mutant, and complement strains were all lysed with EasyLyse™ Bacterial Protein Extraction Solution (Epicentre, Madison, Wisconsin) to release intracellular proteins. The supernatant was separated from bacterial debris by centrifugation; protein concentration was then measured by BCA Protein Assay Kit (Pierce®, Rockford, USA). GDH activity of 100 μg of *B. pseudomallei* proteins, wild type K96243, SDO mutant, and complement, were determined in a microtiter plate using the GDH Activity Assay Kit (BioVision, Mountain View, USA) as described by the manufacturer. 100 μl of reagent mixture containing assay buffer, developer, and glucose was briefly mixed with 50 μl of protein samples, reagent control, or GDH positive controls. The OD values at 450 nm of the mixtures were measured before and after incubating for 1 hr at 37°C. The NADH standard curve was constructed to determine GDH activity (mU/mg).

### Phenotypic examination of the *B. pseudomallei* SDO mutant

Colony morphology of the *B. pseudomallei* SDO mutant on Ashdown agar at day 4 was examined using a morphotyping algorithm [26]. Bacterial structure was determined under light microscopy (Gram stain) and electron microscopy. The ability of the *B. pseudomallei* SDO mutant to invade A549 cells and survive in infected J774A.1 cells was measured as previously described [51], and compared with the wild type strain.

In the invasion efficiency assay, an A549 cell line was infected with culture of *B. pseudomallei* in LB broth containing 0, 150, or 300 mM NaCl at a multiplicity of infection (MOI) of 100 for 1 hr to bring bacteria into contact with the cells and allow bacterial entry. The monolayers were overlaid with a medium containing 250 μg/ml kanamycin (Gibco) to kill extracellular bacteria for 1 hr. Viable intracellular bacteria were released from the infected cells at 4 hrs post-infection by lysis with 0.5% Triton X-100 (Sigma-Aldrich), and then plated on Trypticase soy agar. Colony forming units were measured after 36–48 hrs of incubation at 37°C. The percentage of invasion efficiency is calculated as the number of intracellular bacteria at 4 hrs post-infection × 100 and divided by the CFU added.

For the intracellular survival assay, a J774A.1 cell line was inoculated with culture of *B. pseudomallei* in LB broth containing 0, 150, or 300 mM NaCl at a multiplicity of infection (MOI) of 2 for 2 hrs to allow bacterial entry. After infection for 2 hrs, a medium containing 250 μg/ml kanamycin was added to kill extracellular bacteria. The cell culture was incubated for 2 hrs to completely eliminate residual extracellular bacteria. An additional incubation was then performed; infected cells were covered with a medium containing 20 μg/ml kanamycin to inhibit the growth of the remaining extracellular bacteria. After 4, 6, and 8 hrs post-infection, the cell monolayer was washed with pre-warmed PBS and lysed with 100 μl of 0.1% Triton X-100 (Sigma Chemical Co.) in distilled water. Intracellular bacteria were quantitated by dilution and plated on Trypticase soy agar. The bacterial colonies were counted after 36 hrs of incubation at 37°C. The percentage of intracellular survival was determined by the following equation: (number of intracellular bacteria post-infection × 100)/ number of CFU added.

### Determination of the *B. pseudomallei* survival under oxidative stresses

The survival of *B. pseudomallei* in oxidative conditions was determined by the growth on oxidant agar plates. The 6 hrs cultures of *B. pseudomallei* in LB broth containing 0, 150, or 300 mM NaCl were washed and resuspended with PBS. The bacterial concentration was adjusted to OD_600_ 0.15. A 10-fold dilution of the inoculums was performed. Ten microlitres of all dilutions of bacteria in PBS were spotted onto the LB agar with and without adding sub-lethal concentrations of menadione (400 μM), H_2_O_2_ (250 μM) and tBOOH (200 μM) [52]. Colony counts were performed after incubation at 37°C for 24 hrs. The number of colonies on plates containing oxidants was compared with that on control plates (LB agar without oxidant) and presented as % bacterial survival. % Survival = CFU (with oxidant) × 100/ CFU (without oxidant).

### Statistical analysis

All assays were conducted in triplicate, and unpaired *t*-test of independent experiments was performed by statistical analysis using GraphPad Prism 6 program (STATCON). Results were considered significant at *p*-value ≤ 0.05.

## Competing interests

The authors declare that they have no competing interests.

## Authors’ contributions

PP and NC designed the research. UB performed bioinformatics analysis of protein sequences. PP and ST constructed the mutant. PP and PP constructed the complementary strain. PP and BU carried out enzymatic activity assay. PP and VM carried out growth kinetic assay. PP and TS performed the colony examination. PP and PP carried out invasion and survival assay. PP and MV performed the oxidative response experiment. PP carried out statistical analysis. PP wrote the manuscript. NC and KS critically revised the manuscript for intellectual content. All authors read and approved the final version of the manuscript.

## Supplementary Material

Additional file 1 **Construction and verification of *B. pseudomallei *** **SDO mutant. A)** A 566 bp DNA fragment containing 298 bp-upstream and 288 bp-downstream of the SDO gene was replaced into the *B. pseudomallei* K96243 genome using the pEXKm5-based allele replacement system [19]. **B)** PCR of *B. pseudomallei* wild type, SDO mutant and SDO complement strain were performed with the BPSS2242-F1 and BPSS2242-R2 primer pair (lane 1: 100–3000 bp marker ladder; lane 2: negative control; lane 3: K96243; lane 4: SDO mutant; and lane 5: SDO complement strain). **C)** PCR analysis of pEXKm5 plasmid backbone within the *B. pseudomallei* genome using oriT specific primers (lane 1: 100–3000 bp marker ladder; lane 2: negative control; lane 3: SDO mutant before sucrose selection; lane 4: SDO complement strain before sucrose selection; lane 5: SDO mutant after sucrose selection; and lane 6: SDO complement strain after sucrose selection). Click here for file
