# Supplementary figures and images for: The role of short-chain dehydrogenase/oxidoreductase, induced by salt stress, on host interaction of B. pseudomallei
Source: BMC Microbiol. 2014 Jan 2;14:1. doi: 10.1186/1471-2180-14-1 (PMC3882111; doi:10.1186/1471-2180-14-1)

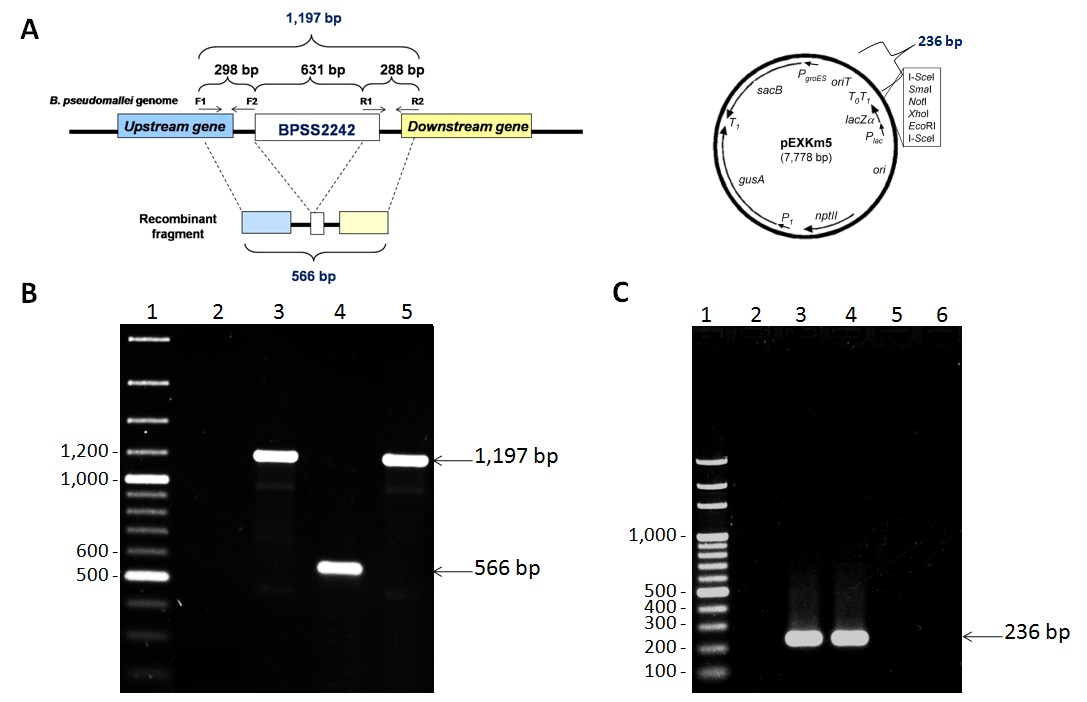

Supplement: Additional file 1 — Construction and verification of B. pseudomallei SDO mutant. A) A 566 bp DNA fragment containing 298 bp-upstream and 288 bp-downstream of the SDO gene was replaced into the B. pseudomallei K96243 genome using the pEXKm5-based allele replacement system [19]. B) PCR of B. pseudomallei wild type, SDO mutant and SDO complement strain were performed with the BPSS2242-F1 and BPSS2242-R2 primer pair (lane 1: 100–3000 bp marker ladder; lane 2: negative control; lane 3: K96243; lane 4: SDO mutant; and lane 5: SDO complement strain). C) PCR analysis of pEXKm5 plasmid backbone within the B. pseudomallei genome using oriT specific primers (lane 1: 100–3000 bp marker ladder; lane 2: negative control; lane 3: SDO mutant before sucrose selection; lane 4: SDO complement strain before sucrose selection; lane 5: SDO mutant after sucrose selection; and lane 6: SDO complement strain after sucrose selection). [file 1471-2180-14-1-S1.tiff]
